# Community-Based Monitoring: Key to Success of National Health Programs

**DOI:** 10.4103/0970-0218.66857

**Published:** 2010-04

**Authors:** Suneela Garg, Ananya Ray Laskar

**Affiliations:** Department of Community Medicine, MAMC, New Delhi, India

The National Rural Health Mission (NRHM) was launched on April 12, 2005 by the UPA Government with the goal of improving the availability of and access to quality healthcare by people, especially for those residing in rural areas, the poor, women and children. In order to ensure that the services reach those for whom they are meant, the NRHM proposes an intensive accountability framework that includes Community-based Monitoring (CBM) as one of its key strategies.(1)

So one of the prerogatives of the Government of India is to assess whether the national health programs under NRHM are able to achieve the targeted goals and meet the timelines. Looking at the quantum of these programs, the existing monitoring systems are inadequate to bring out optimal trend analysis of key performance indicators. Answers to these questions lie in community monitoring through involvement of local beneficiaries.(2) At the moment, the monitoring of Health Programs suffers from numerous setbacks such as no dedicated cell for CBM at state or district level and lack of standardized reference manual for planning and monitoring. Multiplicity of registers and duplication of reports are still a common practice. Voluminous data are collected, which is rarely validated or used in planning. There is a mismatch between routine MIS and survey data.

The 73^rd^ and 74^th^ amendments to the Constitution of India (April 1993) reiterates Government attempts to institutionalize CBM in health on a larger scale. Above all it is consistent with the right to health care approach till the grassroot level.(3)

## CBM: The Concept

Community-based Monitoring involves drawing in, activating, motivating, capacity building and allowing the community and its representatives e.g. communitybased organizations (CBOs), people’s movements, voluntary organizations and Panchayat representatives, to directly give feedback about the functioning of public health services.(4) The community monitoring process will involve a three-way partnership between healthcare providers and managers (health system); the community, community-based organizations, NGOs and Panchayati Raj Institutions. The emphasis will be laid on the developmental spirit of ‘fact-finding’ and ‘learning lessons for improvement’ rather than ‘fault finding’.(2)

Stages of CBM:Community-based monitoring process includes preparatory activities, capacity building and training of trainers, community assessment, interface meeting and finally the evaluation.

## The Following Five Stages are Involved in the Community Monitoring Process

### Stage I

Preparatory activities (Identification of Stakeholders, levels of services for community monitoring):

- Constitution of a task force group by inclusion of representatives from the coordinating agency, state policy makers and civil society members for planning, designing, advising and overall monitoring of the community process.
- Community monitoring teams should be tailor-made according to the programs at the different levels of healthcare services in a sequential manner. These should have predefined criteria for eligibility for inclusion as team members so as to have an equal representation of all users.

### Stage II

Training of stakeholders is imperative for effective community monitoring, capacity building of beneficiary representatives, community-based organizations (CBOs), NGOs, voluntary organizations and panchayat representatives, who will eventually be providing the feedback.

Accountability building, health rights with clarity of role and concomitant authority should be well-defined at the outset of the assessment process. NGOs and CBOs would contribute to the collection of information relevant to the monitoring process at all the levels from the village to the state level. (included in the manual for CBM under NRHM).

Focus of training should be at the district level since the district is the unit to be strengthened. District level workshop should be held to share the concept, identify blocks and PHCs, involving key district health officials, PRI members and civil society organizations. The CBOs should monitor demand, coverage, access, quality, effectiveness, behavior and presence of healthcare personnel at service points, possible denial of care and negligence. All the above monitored should be related to outreach services, public health facilities and referral system.

### Stage III

Community Assessment (Development of tools and Techniques – In-depth Interviews, FGDs, Case Studies, Record Review, Citizens Report Card)

It is a CBM tool that is a hybrid of the techniques of social audits and citizen report cards.

- Community score card (CSC): is an instrument to achieve social and public accountability and responsiveness from service providers. By linking service providers to the community, citizens are empowered to provide immediate feedback to the providers.
- Report Card: is a technique used to generate public feedback on the degree of citizen’s satisfaction with the quality of service provided by public agencies. It serves as a diagnostic tool for service providers and others to identify problem spots or deficient areas that need attention within an agency. It also helps to encourage public agency to initiate consumer friendly practices and policies, internal performance measures and increased transparency in operations. It has three colour codes on the basis of the progress of the various activities.

Green = 75–100% activities completed or on track.

Yellow = 50–74% activities completed or on track.

Red = 1–49% activities completed or on track.

### Stage IV

Interface Meeting (Collection of data/Feedback and dialogue) PHC and Block level community monitoring exercises should include a public dialogue ‘Jan Samvad’ or public hearing ‘Jan Sunwai’. Here individual testimonies and assessments by local CBOs/NGOs would be presented. These would be facilitated by the district and block facilitation groups in collaboration with panchayat representatives and CBOs/NGOs working on the issue of health rights.

### Stage V

Evaluation of feedback (Data entry, Data analysis and report submission and review, documentation). Data collected should be complied, collated and analysed in a standardized manner at different levels depending upon the availability of service so as to aggregate data and also have specific information about the individual service.

**Figure 1:**
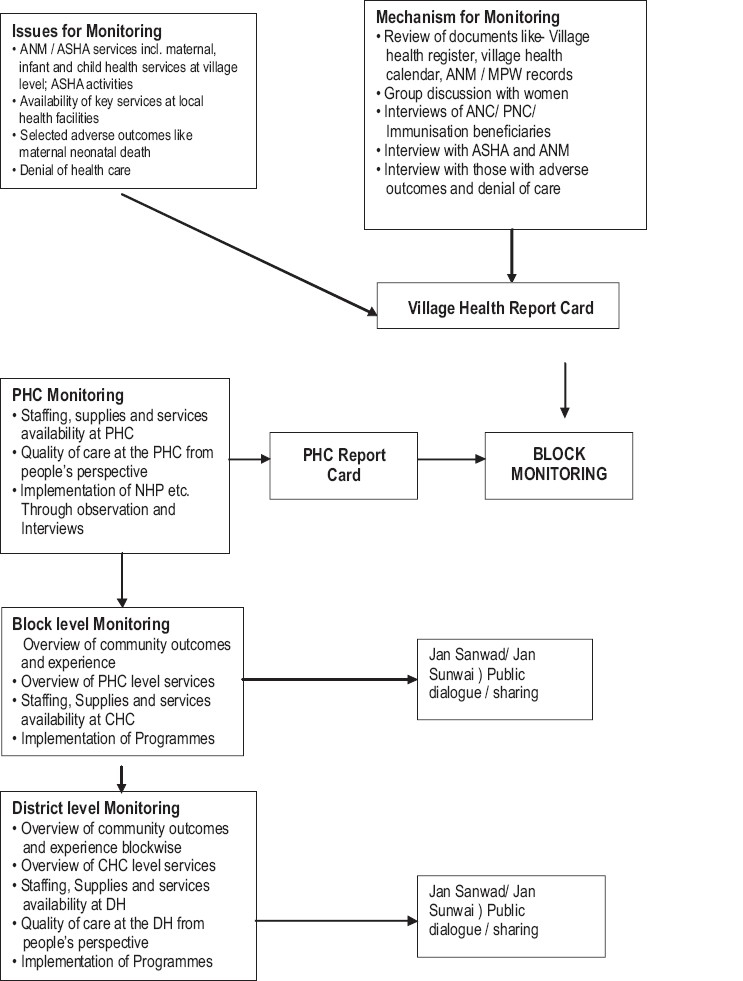
The process of monitoring

Members of the task force should monitor the implementation in the field through frequent, regular and planned visits. The proposed flow of monitoring at different levels is shown in Figure 1. In a nutshell, CBM entails formation of planning and monitoring committees at the level of village, PHC and block. Each of these committees would have representation from service providers, Panchayati Raj Institutions, community and civil society organizations. The committees send a periodic report (Quarterly at the village, PHC, block or district level and six monthly for state level) to the next higher level committee.

## Current Status of CBM

Community-based Monitoring is still an evolving concept, currently being piloted in nine states of India (Assam, Chhattisgarh, Jharkhand, Karnataka, Madhya Pradesh, Maharashtra, Orissa, Rajasthan and Tamil Nadu). In each state, 3–5 districts have been chosen depending on the number of districts. Three blocks in each district, three PHCs in each block and five villages in each PHC have been chosen for this phase. Workshops have been held for scaling up of community monitoring and popularize it.(5) However, more sensitization and sustained efforts are required for integration and institutionalization of CBM in order to fulfill the goals of NRHM.

The program can learn lessons from other projects, which are currently implementing this as a part of their projects such as ‘Swasth Plus’ - Community Monitoring Project, Karnataka; People’s Health Management Information System Project, Orissa; ‘Prayas’, Rajasthan; Rural Poverty Reduction Project (Monitoring of Health Services), Andhra Pradesh(4,6) etc. The common reasons behind the success of CBM in these projects are strong presence of civil societies, involvement of public health personnel as well as the community as principle stakeholders, adequate geographic representation and crucial role by monitoring committees. Monitoring committees here not only review and collate the records of the subunits but also appoint a small subteam from its NGO who visit a small sample of units (one facility and two villages) under their purview.

## Potential strengths of CBM

So CBM of health services is a key strategy of National Rural Health Mission to ensure that the services reach to those for whom they are meant. This framework is consistent with the ‘Right to Health Care’ approach since it places health rights of the community at the center of the process. It seeks to address the gaps in the implementation of various programs and thereby enhancing the transparency till the grassroot level. It is also being seen as an important aspect for promoting community-led action.
